# Origins of cancer symposium 2016: exploring tumor complexity

**DOI:** 10.18632/genesandcancer.119

**Published:** 2016-09

**Authors:** Matt Harlow, Kwang-Ho Lee, Mary Winn

**Affiliations:** ^1^ Department of Cancer Biology, Vanderbilt University, Nashville, TN, USA; ^2^ Center for Epigenetics, Van Andel Research Institute, Grand Rapids, MI, USA; ^3^ Bioinformatics and Biostatistics Core, Van Andel Research Institute, Grand Rapids, MI, USA

**Keywords:** tumor complexity, tumor evolution, therapy resistance, mediator, review

## Abstract

Cancer has challenged researchers with its immense complexity, from initiation to progression and on to therapeutic resistance. The seventh Origins of Cancer Symposium, held on July 22, 2016, at Van Andel Research Institute, was organized around the theme “Exploring Tumor Complexity”, and the latest advances under that theme from seven leading cancer research laboratories were discussed. Here we summarize highlights from the meeting and their implications.

## INTRODUCTION

The symposium began with a presentation on intratumor heterogeneity, an emerging aspect of tumor complexity. Nicolas Navin (MD Anderson Cancer Center) shared important and novel insights into how intratumor heterogeneity influences tumor evolution and therapy resistance. Using single-nucleus sequencing, Dr. Navin discovered that aneuploid rearrangements occur early in breast tumor evolution and remain highly stable during subsequent clonal expansions [1]. This observation challenges the paradigm that copy number alterations (CNAs) occur gradually and continuously during the course of tumor progression [2, 3]. His laboratory also discovered that, unlike CNAs, point mutations evolved continuously over time, leading to extensive clonal diversity, i.e., a large number of rare subclonal (<1%) mutations that may play an important role in tumor evolution and therapy resistance [4]. Dr. Navin also discussed cancer mutation rates. His group found the mutation rate of an ER+ breast tumor was similar to that of normal cells, whereas triple-negative breast cancer showed a mutation rate 13.3× higher [4]. These rates are substantially lower than previous estimates from bulk tumor samples [5]. Lastly, Dr. Navin demonstrated how phylogenetic trees of each tumor constructed by single-cell sequencing methods can guide targeted therapy by revealing founder mutations in the “trunk” of the tree; such founders can serve as ideal therapeutic targets because they are shared by all cells in the tumor.

Sophia Lunt (Michigan State University) switched gears toward cancer metabolism and discussed how aberrant alterations in metabolic regulators contribute to cancer progression and metastasis. Dr. Lunt put forward a proposition that the major function of enhanced aerobic glycolysis in cancer cells is to maintain high levels of glycolytic intermediates to promote cell proliferation [6]. As evidence, Dr. Lunt showed that an increase in glycolytic metabolites through inhibition of a pyruvate kinase muscle isozyme (PKM2), a key regulator of glycolysis, enhanced tumor cell proliferation [7]. This suggests that high pyruvate kinase activity may suppress tumor growth. Indeed, she went on to show that both PKM2 activation and the expression of PKM1, an isoform with high constitutive kinase activity, inhibit the growth of xenograft tumors [8]. Dr. Lunt then demonstrated that the anti-proliferative influence of high PKM activity holds true for normal cells as well by showing that PKM1 expression in primary normal cells impaired nucleotide production, leading to proliferation arrest [9]. Dr. Lunt concluded the talk with her findings on the role of SDHB, a subunit of succinate dehydrogenase, in ovarian cancer [10]. Targeted knockdown of *Sdhb* in mouse ovarian cancer cells resulted in enhanced proliferation and an epithelial-to-mesenchymal transition (E-MT) mediated by a genome-wide increase in histone methylation [10]. This work provides an insight into how *SDH* dysfunction can promote ovarian cancer progression.

The second session of the symposium was focused on the complexities that underlie the process of transcription and novel methods for specifically targeting this process for cancer therapy. The first speaker was Dr. Dylan Taatjes (University of Colorado - Boulder) and his talk focused on the Mediator complex. Dr. Taatjes provided an in-depth review of what is known about the role of Mediator in transcription, with a specific focus on how this protein complex relays signals from specific transcription factors to the polymerase machinery. The latter half of his seminar detailed the efforts that have gone into drugging the Mediator complex, including one of his recent papers that identified cortistatin A as a highly specific inhibitor of CDK8 [11]. Intriguingly, they found that acute myeloid leukemia (AML) cells are highly sensitive to cortistatin A treatment. Dr. Taatjes showed that such treatment up-regulated tumor-suppressor and cell-lineage genes, and that this increase exposed a vulnerability of AML cells to the dosage of superenhancer-associated genes. In the hopes of translating this compound to the clinic, Dr. Taatjes reiterated that cortistatin A had no generally cytotoxic effects *in vivo.* Dr. Taatjes concluded his talk by describing recent efforts to identify substrates of CDK8, using SILAC followed by mass spectrometry [12].

The fourth speaker of the day was Dr. Rani George (Dana-Farber Cancer Institute, Harvard). Dr. George's lab is focused on neuroblastoma (NB), and her talk centered on the role of *CDK7* in this tumor. Dr George began by outlining the differences between cell-cycle CDKs and transcriptional CDKs. She followed with an elegant portrait of the NB field and where it stands with regard to prognostic markers and treatment options. Dr. George stressed that NB patients that have increased expression of the MYCN protein have the poorest prognosis [13]. Interestingly, the amplification of MYCN protein in NB results in MYCN accumulation at all actively transcribed genes and causes global amplification of transcription [14]. Dr. George then showed that *MYCN* expression and global transcription amplification are reduced by *CDK7* knockdown. Because of the recent characterization of THZ1 as a small molecule that irreversibly inhibits CDK7 activity, Dr. George's group sought to describe the effect of THZ1 on MYCN-amplified cells. THZ1 treatment caused a marked decrease in MYCN expression and a global decrease in transcription, but only in cells with increased MYCN expression. Dr. George identified a unique sensitivity in MYCN-amplified NB cells that correlated with the down-regulation of oncogenic driver genes regulated by superenhancers, including *MYCN*. Importantly, the effect of THZ1 on transcription was to suppress MYC-driven transcriptional programs while sparing other transcriptional programs. In agreement with this observation, Dr. George noted that there was no observable off-target toxicity of THZ1 when administered to mice *in vivo*.

In the next talk, Emily Bernstein (Icahn School of Medicine at Mount Sinai) introduced attendees to the role of histone variants in cancer initiation and progression, with a particular focus on macroH2A. This histone, when incorporated into the nucleosome, serves as a transcriptional repressor. As such, Dr. Bernstein showed that macroH2A serves as repressor of melanoma progression [15, 16]. This led to interesting work studying the role of macroH2A in pluripotency and reprogramming, which are important features of malignant melanoma. Using macroH2A-deficient mice (dKO), Dr. Bernstein showed macroH2A serves as an epigenetic barrier to reprogramming [17]. Intriguingly, dKO females have delayed mammary development, and this finding has led to the study of macroH2A in mammary development and breast cancer pathogenesis. Early findings have implicated macroH2A in the maintenance of differentiated mammary cell populations in dKO mice, while macroH2A levels are decreased in invasive breast cancer. Together, these findings further highlight the importance of epigenetic regulation in the origin and progression of cancer.

David Langenau (Massachusetts General Hospital, Harvard), provided a detailed mechanistic view of how T-cell acute lymphoblastic leukemia (T-ALL) progresses and develops resistance to a therapy. Using an elegant approach of single T-ALL cell transplantation into zebrafish, his lab was able to follow the clonal evolution process of T-ALL. They observed functional variation within individual clones and found that a minority of clones gained an enhanced growth rate and leukemia-propagating potential by mTORC1 activation mediated through Akt pathway activation. Akt activation was also found to render cells resistant to dexamethasone. They found that this resistance was reversible by combined treatment with an Akt inhibitor. He then shared unpublished data on a putative oncogene, *TOX* (thymocyte selection-associated high mobility group box), that they recently identified through a transgenic screening approach. They found that TOX exacerbates the onset of leukemia when co-overexpressed with MYC and that TOX is overexpressed in 95% of human T-ALL by a superenhancer containing TAL1 and MYB. This high incidence suggests that the overexpression of TOX might be an initiating event of T-ALL. He then presented evidence that TOX exerts its oncogenic function by inducing genomic instability through acting as a negative regulator of KU70/80.

Susan Rosenberg (Baylor College of Medicine) brought the day to a close by bringing an evolutionary biology perspective to the regulation of mutagenesis in cancer cells. Challenging a model of random mutagenesis, Dr. Rosenberg presented work implicating stress and genomic location as nonrandom mechanisms of mutagenesis using *E. coli* as a model. Specifically, three stress responses regulate mutagenesis — the starvation or general stress response/RpoS [18], the SOS DNA damage response [19], and membrane protein stress or UPR [20] — through mutagenic break repair [21-23]. Dr. Rosenberg has shown that one mechanism responsible for mutagenic break repair is the switching from high-fidelity to error-prone double-strand-break repair through the polymerase DinB [24]. Further work has shown that the stress response is necessary to induce mutagenesis in the presence of double-strand breaks [25]. Further, mutation clusters localize to double-strand break sites [26], with the potential for mutations to occur at actively transcribed regions and transcriptional R-loops [27]. Overall, mutagenic break repair is regulated by a large network of genes [28] and is a common theme among yeast, flies, humans, and cancer cells. New findings are implicating drugs, particularly those that induce double-strand breaks, in the evolution of mutations, sending us home with the message that we need to hit the process (i.e., mutagenic stress response)—rather than the products of evolution— in order to overcome drug resistance.

This year's Origins of Cancer symposium was organized around the concept that cancer is not a simple disease that can be addressed with a single solution. With that in mind, the symposium highlighted three separate but interconnected topics that capture the complexity inherent in cancer. While the themes of gene regulation, tumor heterogeneity, and tumor evolution seem disparate at the surface, the convergence of these topics has advanced our understanding of how tumors function. From these novel discoveries, new vulnerabilities might be identified and exploited therapeutically for clinical benefit.
